# Integrative Review of Family Health Nursing Support for Single-Parent Families: Evidence Gaps and Implications for a Relational Empowerment Model

**DOI:** 10.3390/healthcare14081088

**Published:** 2026-04-20

**Authors:** Elisabete da Luz

**Affiliations:** Escola Superior de Enfermagem, Universidade de Lisboa, 1600-096 Lisboa, Portugal; el@enfermagem.ulisboa.pt

**Keywords:** family nursing, single-parent families, family-centred care, primary health care, empowerment, resilience

## Abstract

**Highlights:**

**What were the main findings?**

- Single-parent families are a prevalent and vulnerable family structure, yet nursing interventions tailored to their needs remain underexplored.
- Effective interventions included psychosocial support, empowerment-focused care, telehealth, and the use of family nursing models such as the Calgary framework, the Family Resiliency Model, and Family Health Conversations.

**What are the implications of main findings?**

- Practice: Nurses should design care pathways that integrate empowerment strategies and adapt to single-parent contexts. Policy: Health systems should allocate resources to targeted support for single-parent families.
- Research: More studies are required to assess effectiveness of interventions across cultures. Education: Nursing curricula should emphasize capacity-building in family-focused interventions for single-parent families.

**Abstract:**

**Background/Objectives:** Single-parent families represent a growing and particularly vulnerable family structure within community and primary health care contexts. These families often experience cumulative burdens related to caregiving overload, socioeconomic constraints, social isolation, and fragmented support networks, which directly affect health and well-being. This integrative review aimed to synthesize and critically analyse direct and conceptually transferable evidence relevant to Family Health Nursing interventions supporting single-parent families in community and primary health care contexts, identify existing knowledge gaps, and inform the development of a relational empowerment model. **Methods:** An integrative literature review was conducted following PRISMA 2020 guidelines. A comprehensive search was performed across three electronic databases (PubMed, CINAHL, and Scopus) covering publications from 2020 to 2025. Inclusion criteria comprised peer-reviewed empirical studies and reviews addressing nursing or health interventions relevant to single-parent families in community or primary health care contexts. Data were extracted and synthesized thematically, with attention to theoretical frameworks, intervention characteristics, and reported outcomes. **Results:** Twenty-nine studies met the inclusion criteria. The synthesis revealed four main thematic domains: (1) caregiving burden and psychosocial vulnerability, (2) access to and coordination of community-based resources, (3) nurse–family relational processes, and (4) empowerment-oriented nursing interventions. Theoretical underpinnings frequently included family systems perspectives, the Calgary Family Assessment and Intervention Models, and empowerment-oriented frameworks. **Conclusions:** Nursing interventions for single-parent families in community health settings should prioritise relational empowerment approaches that acknowledge family diversity, contextual vulnerability, and dynamic caregiving demands. The proposed relational empowerment model offers a practice-informed framework to guide Family Health Nursing interventions, education, and policy development, supporting more responsive and equitable care for single-parent families.

## 1. Introduction

Social changes in recent decades—such as increased acceptance of divorce, women’s autonomy, diversification of family configurations, and evolving gender roles—have contributed to the growing prevalence of single-parent families. These families may emerge from marital dissolution or from individual choices supported by legal, social, and technological advances. Single parenthood entails specific structural and relational characteristics that significantly shape health–illness processes and care needs.

Evidence consistently shows that single-parent families face heightened challenges, including socioeconomic vulnerability, parental overload, social isolation, and barriers in accessing health services, all of which negatively affect well-being and quality of life. In this context, Family Health Nursing (FHN) plays a central role in identifying vulnerabilities and implementing family-centered interventions that promote empowerment and resilience. Despite this relevance, research specifically addressing Family Health Nursing interventions for single-parent families in primary health care remains scarce and fragmented, limiting the responsiveness of health systems to this growing family group.

Community and family nursing practice has traditionally focused on maternal–child health, often privileging the mother–infant dyad. This reductionist approach has contributed to the under-recognition of the relational, emotional, and social complexity of single-parent families, limiting the development of comprehensive family-centred interventions.

Systemic and family nursing theories offer a robust framework to address these gaps. The Calgary Family Assessment Model enables a comprehensive appraisal of family structure, development, and functioning, supporting tailored intervention planning. Meleis’ Transitions Theory conceptualizes single parenthood as a situational and health–illness transition, characterized by both vulnerability and potential for growth. Complementarily, empowerment-oriented approaches emphasize autonomy, self-management, and active participation of families in their care processes.

Although these theoretical frameworks are well established, evidence regarding their application and effectiveness in interventions targeting single-parent families within primary health care remains dispersed. An integrative synthesis of recent literature is therefore warranted to identify key challenges and effective Family Health Nursing interventions for this population [1]. Single-parent family structures present specific characteristics that strongly shape health and illness processes, as well as care needs and follow-up requirements [2,3,4].

Recent studies indicate that single-parent contexts are often associated with increased challenges such as economic vulnerability, parental overload, social isolation, and difficulties in accessing health services-factors that directly affect family quality of life and well-being [5,6,7,8].

Single-parent families are likely to experience higher levels of stress and burden, coupled with limited support from the extended family and ineffective intra-family communication, which may lead to emotional distress and difficulties in self-managing health. In this review, a single-parent family is defined as a family structure in which one adult assumes sole parental responsibility for one or more children [1].

Family Health Nursing plays a crucial role in identifying family vulnerabilities and in developing interventions that promote empowerment and resilience in single-parent families [9,10,11,12,13,14]. However, the literature focusing specifically on Family Health Nursing interventions for single-parent families in community settings remains relatively scarce and fragmented [6]. This gap can be attributed to structural and methodological factors. Historically, community and family nursing practice has centered primarily on maternal–child care, often focused on the mother–infant dyad, neglecting a systemic and integrative approach to the family as a unit of care. This reductionist view has obscured the relational, emotional, and social complexity that characterizes these families, limiting the development of more comprehensive interventions that respond to their realities [15].

Understanding single parenthood requires a systemic family perspective. The Calgary Family Assessment Model (CFAM), widely used in Family Health Nursing practice, enables a comprehensive appraisal of family structure, development, and functioning, facilitating tailored intervention planning [7,16].

Complementarily, Meleis’ Transitions Theory offers insight into changes associated with marital breakdown, widowhood, single-parent decision-making, health–illness transitions, and situational transitions. These circumstances are recognized as life transitions with the potential to generate crisis as well as growth, where nurses can intervene to facilitate healthy transitions [17]. Empowerment-oriented approaches are also relevant to supporting single-parent families by promoting autonomy and the ability to manage chronic illness [18]. Recent evidence emphasizes the importance of interdisciplinary strategies, with a focus on continuity of care, active listening, and therapeutic relationships that respect diversity and promote equity in health care [19].

Operating in community contexts, family nurses are well positioned to implement preventive, educational, and psychosocial support interventions that address the unique needs of single-parent families throughout the life cycle [13,20].

Despite growing recognition of the specific needs of single-parent families, evidence on Family Health Nursing interventions targeting this group remains scarce and fragmented, limiting the responsiveness and effectiveness of primary health care services.

## 2. Methods

The purpose of this integrative review was to explore the scientific literature published between 2020 and 2025 on the challenges faced by single-parent families in health care contexts and the Family Health Nursing interventions implemented to support them, based on studies conducted in primary health care settings. The increasing prevalence of single-parent families, together with their specific needs and social vulnerabilities, highlights the relevance of this topic for the development of specialized practices in Family Health Nursing. This review was not prospectively registered. Nevertheless, it followed established methodological guidelines for integrative reviews and adhered to PRISMA 2020 reporting standards.

### 2.1. Design

Given the scarcity and fragmentation of literature on single-parent families within the scope of Family Health Nursing, an integrative literature review was selected as the most appropriate methodological approach. This method enables the inclusion and critical synthesis of diverse types of evidence—quantitative, qualitative, and conceptual studies—providing a comprehensive understanding of complex phenomena such as single parenthood in family nursing practice.

An integrative review captures the heterogeneity of family experiences by considering the varied configurations and dynamics present in single-parent households. It also allows the analysis of how Family Health Nursing interventions have been developed and implemented in community and primary health care contexts, identifying effective strategies and areas requiring further attention. By integrating different types of evidence, this review contributes to a stronger knowledge base that can guide clinical practice, professional training, and health policy development aimed at supporting single-parent families.

### 2.2. Data Sources

CINAHL, Scopus, and PubMed were searched for empirical and review studies published between January 2020 and March 2025 in Portuguese, English, or Spanish. Only peer-reviewed articles available in full text were considered. For methodological transparency and reproducibility, the complete revised search strategies for each database are presented in the Supplementary Materials (Supplementary File S1). These include database-specific search strings, Boolean combinations, truncations, and controlled vocabulary (MeSH and CINAHL Headings), reflecting the expanded, sensitivity-enhanced approach adopted in this integrative review.

### 2.3. Review Methods

**Figure 1:**
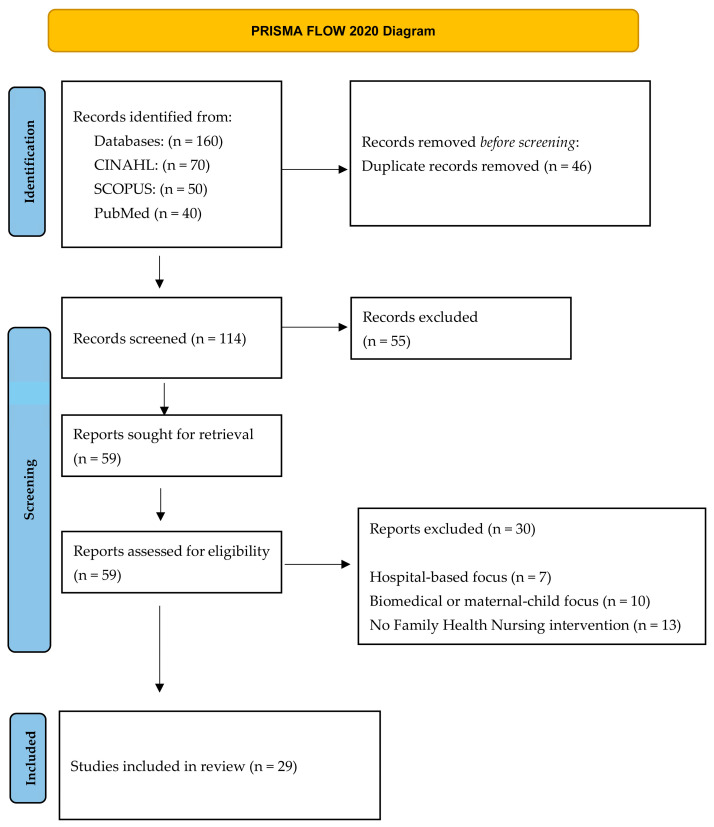
PRISMA 2020 Flow Diagram.

This integrative review was conducted in accordance with PRISMA 2020 reporting guidelines, and the study selection process is presented in Figure 1. The methodological approach followed the framework proposed by Whittemore and Knafl [21], enabling the inclusion and synthesis of evidence derived from diverse methodological traditions, including qualitative and quantitative empirical studies as well as systematic reviews. This approach is particularly suited to complex and underexplored phenomena such as single parenthood within Family Health Nursing practice. Given the limited number of empirical studies explicitly focused on single-parent families within Family Health Nursing and community care contexts, the review adopted an integrative evidence inclusion strategy. In addition to studies directly involving single-parent family samples, research addressing broader family caregiving, vulnerability, and relational health contexts was included when findings demonstrated conceptual and contextual transferability to single-parent family experiences. Transferability was determined based on relevance to relational processes, caregiving demands, resource mobilization, support systems, and empowerment dynamics applicable to single-parent family structures. This methodological decision is consistent with integrative review guidance, which supports the inclusion of diverse evidence sources to generate a comprehensive understanding of emerging or insufficiently studied phenomena. This approach ensured analytical breadth while maintaining the review’s central focus on informing Family Health Nursing support for single-parent families. Study selection and appraisal were conducted by a single reviewer and are acknowledged as a limitation.

The review followed the five structured stages [21]:

1. Identification of the research problem.
2. Definition of inclusion and exclusion criteria.
3. Systematic literature search and selection.
4. Critical appraisal and data extraction.
5. Analysis and synthesis of results.

Methodological guidelines from Souza, et al. (2010) [22] and Mendes, Silveira, et al. (2008) [23] were also applied to ensure rigour and transparency.

Stage 1—Problem Identification

Social and epidemiological changes have significantly influenced family type, structure, and functions. Single-parent families appear to represent one of the family types with the greatest impact on health. This review was guided by the following research questions: How has Family Health Nursing addressed specialized care and support for single-parent families in community/primary health care contexts? What are the main health-related challenges faced by single-parent families?

Stage 2—Inclusion and Exclusion Criteria

Inclusion criteria:

Studies focusing on the family as the unit of care and nursing interventions directed toward single-parent families in community or primary health care contexts. Qualitative, quantitative, or systematic review studies. Peer-reviewed articles with full-text access.

Exclusion criteria:

- Studies focus on hospital-based or institutional care settings (e.g., intensive care units, inpatient hospitalization, long-term residential care), without a community or primary health care perspective.
- Studies centred exclusively on biomedical, maternal, or pediatric clinical outcomes, without a Family Health Nursing or relational caregiving focus.
- Absence of family-focused nursing interventions or relational support components.
- Studies addressing general population health issues without specific reference to family caregiving or family nursing practice.
- Opinion papers, editorials, commentaries, or theoretical reflections lacking empirical or review methodology.
- Articles not available in full text.
- Publications in languages outside the predefined inclusion criteria (English, Portuguese, Spanish).

Studies were excluded only when the primary focus was exclusively biomedical, maternal, or pediatric clinical care, without a Family Health Nursing, relational, or caregiving perspective. Importantly, studies involving children within single-parent family contexts were not excluded when aligned with the relational and family-centred scope of the review. All exclusions were conducted following full-text review in accordance with the predefined eligibility criteria.

Stage 3—Literature Search and Selection

Searches were conducted in the following databases: CINAHL, Scopus, and PubMed. The search period was from January 2020 to March 2025, and studies were considered if published in Portuguese, English, or Spanish. Expanded search strategy (see Supplementary File S1).

The study selection process followed PRISMA 2020 (Figure 1) guidelines, ensuring transparency from identification to final inclusion.

The review followed the five structured stages [21]:

Data extraction was carried out using a structured form capturing the following information for each study:

Transition category [17], Stage of the family life cycle, Challenges identified in single-parent families, reported nursing interventions, Theoretical approach/model applied. The column “Single-Parent Specificity” identifies whether included studies explicitly involved single-parent family samples (direct evidence) or whether findings were considered conceptually transferable from broader family caregiving and vulnerability contexts relevant to single-parent family experiences. The methodological quality of the studies was assessed according to their design, using appropriate appraisal tools for qualitative, quantitative, and review research.

Stage 4—Critical Appraisal and Data Extraction

Methodological guidance [22,23] was also applied to ensure rigour and transparency.

To enhance methodological transparency and rigour, a critical appraisal of the included studies was conducted retrospectively, consistent with integrative review methodological guidance. Given the heterogeneity of study designs, appraisal tools were selected according to methodological approach. The review process was conducted by a single reviewer. To enhance methodological rigour and minimize potential bias associated with single-reviewer screening, a structured and transparent decision-making process was adopted. All records retrieved from the databases were exported to reference management software and screened in two stages: title/abstract screening followed by full-text review.

Eligibility criteria were defined a priori and applied systematically during both stages. When uncertainty regarding study inclusion arose, the reviewer re-examined the eligibility criteria and the full text of the article to ensure consistency with the review objectives. Decisions were documented in a screening log to maintain transparency of the selection process.

Stage 5—Data Synthesis Quality Appraisal

Methodological quality appraisal was conducted using standardized appraisal tools appropriate to the study design. In cases where methodological judgments were uncertain, the reviewer revisited the appraisal criteria and supporting methodological literature to ensure consistency in scoring and interpretation.

Although the review was conducted by a single reviewer, the use of predefined criteria, structured screening procedures, and documented decision-making processes contributed to maintaining methodological transparency and reliability. The appraisal process examined methodological clarity, sampling adequacy, data collection procedures, analytical rigour, and coherence between study aims and findings. In line with integrative review methodology, studies were not excluded based on quality alone; rather, appraisal outcomes were used to contextualize the strength of evidence within the synthesis and to inform interpretation and model development. A summary of the methodological quality appraisal in Supplementary Materials. A summary table of methodological characteristics (Supplementary Table S2) has been added in Supplementary File S2.

The 29 included studies are summarized in Table 1, which highlights transition categories, life cycle stages, challenges, nursing interventions, and theoretical models.

Of the 29 included studies, none explicitly involved samples composed exclusively of single-parent families. All included studies were classified as conceptually transferable based on alignment with structural characteristics of single-parent family dynamics, including concentrated caregiving responsibility, reduced co-parental support, heightened decision-making burden, and relational vulnerability. In this review, the term *transferable evidence* refers to findings derived from studies involving broader family caregiving populations that, although not exclusively focused on single-parent families, describe relational, structural, or caregiving dynamics theoretically applicable to single-parent contexts.

Transferability was considered when study findings addressed vulnerabilities, caregiving responsibilities, resource constraints, or relational processes that are conceptually consistent with challenges commonly described in single-parent family systems. This approach allowed the review to integrate both direct evidence from studies explicitly involving single-parent families and indirect evidence from broader family research that contributes to understanding the contextual and relational dimensions of empowerment in these families.

This finding reinforces the limited availability of single-parent-specific intervention research within Family Health Nursing. Transferable evidence was not weighted statistically; however, interpretive priority was given to findings demonstrating higher contextual alignment with single-parent structural characteristics. These findings formed the basis for thematic synthesis in the results.

## 3. Results

The articles included in this integrative review revealed a wide range of challenges faced by families across different stages of the life cycle, with a particular focus on health–illness transitions and family roles. The identified nursing interventions addressed the specific needs of each family, with a predominance of approaches targeting the affective, behavioural, and cognitive domains, aligned with theoretical frameworks and the capacity-building of health professionals for Family Health Nursing interventions. The challenges identified were categorized as follows:

1. Caregiver Burden
2. Socioeconomic vulnerability
3. Social isolation and lack of support networks
4. Intra- and extra-family communication challenges

Challenges derived from transferable family caregiving were the most frequently reported category in the articles, with specific patterns emerging in nursing interventions for single-parent families experiencing such transitions. Care strategies were adapted to the health condition of the family member.

Another important aspect highlighted was the family role transition, which significantly affects family structure, development, and functioning—such as cases in which grandparents assume parental responsibilities in place of parents. Several articles indicated that one of the main challenges for single-parent families during health–illness transitions is the emotional and physical overload experienced by caregivers. Studies involving families affected by cancer, schizophrenia, and dementia consistently reported high levels of stress and exhaustion among caregivers [4,5,24]. Lack of social support and communication difficulties within the family were identified as factors that exacerbate these challenges [8,24,25].

Moreover, families in vulnerable situations—such as homeless families in parenting contexts and LGBTQ adoptive families experiencing disrupted placements—faced additional barriers, including housing insecurity, stigma, and unacknowledged grief, which hindered transition and family reorganization [26,27]. These factors were further intensified by financial constraints and social stigma, imposing a significant burden on family members.

The column “Single-Parent Specificity” identifies whether included studies explicitly involved single-parent family samples (direct evidence) or whether findings were considered conceptually transferable from broader family caregiving and vulnerability contexts relevant to single-parent family experiences.

### Nursing Interventions

According to the analyzed articles, nursing interventions varied considerably depending on the family’s specific circumstances. Most studies emphasized the importance of the affective and behavioral domains, particularly in families coping with the loss of a loved one or living with severe chronic illness. The use of family-centered care models, such as the Family-Centered Care Pathway and the Family Resiliency Model, was reported as effective in promoting family resilience and facilitating adaptation to new realities [3,5]. Capacity-building models for nurses, including training in Family Nursing Conversations and culturally responsive home-visiting approaches, were highlighted as essential to improve communication with diverse families and to provide more accessible and effective care [28,29]. In families facing chronic illness or end-of-life situations, such as those with members undergoing oncology treatment or living with Alzheimer’s disease, interventions focusing on the cognitive domain—such as stress management and psychosocial support—were particularly important [24,30,31]. Caregiver support interventions and strategies to strengthen families’ internal resources were also found to mitigate the negative impacts of illness [31,32]. Models such as the Calgary Family Assessment Model and the Family Management Framework provide a robust theoretical structure for assessing family functioning and guiding tailored interventions [8,16]. Overall, nursing interventions were varied and adapted to the needs and characteristics of families in transition. A culturally sensitive approach and personalized care planning also appeared fundamental for effective intervention in families facing vulnerability.

Across the included studies, empowerment emerged as a cross-cutting outcome of Family Health Nursing interventions, regardless of family life cycle stage or health–illness context. Empowerment was reflected in increased caregiver self-efficacy, enhanced capacity for decision-making, improved access to community resources, and greater involvement of families as active partners in care planning. Interventions grounded in systemic and strengths-based models were particularly associated with positive empowerment-related outcomes.

## 4. Discussion

A central finding of this integrative review is the limited body of empirical evidence specifically addressing Family Health Nursing interventions targeted at single-parent families. Despite the growing prevalence and recognized vulnerability of single-parent households, the literature remains predominantly focused on broader family caregiving contexts, illness-related transitions, and general family support interventions. This scarcity required the inclusion of conceptually transferable evidence to inform relational and empowerment processes relevant to single-parent family experiences. While such transferability enabled analytical depth, it also underscores a critical gap in the development and evaluation of targeted nursing interventions for this population. The inclusion of conceptually transferable evidence allowed the review to capture broader relational and structural mechanisms influencing family empowerment, while acknowledging that empirical studies specifically focusing on single-parent families remain limited. The absence of single-parent-specific intervention studies has important implications for Family Health Nursing practice and research. It highlights the need for the design, implementation, and evaluation of relational, community-based, and empowerment-oriented nursing approaches tailored to the unique structural, emotional, and socioeconomic realities of single-parent families. Addressing this gap represents a strategic priority for advancing equitable, family-centered primary health care and strengthening the evidence base underpinning specialized Family Health Nursing practice. Importantly, nurses should move beyond focusing solely on vulnerabilities to also recognize resilience, intergenerational solidarity, and community resources. The analysis of the 29 studies resulted in three main thematic axes:

### 4.1. Expanded Needs of the Sole Caregiver

In health–illness transitions, the single-parent caregiver accumulates multiple roles—provider, care manager, and emotional support—thereby intensifying the emotional and physical burden already documented in broader family caregiving contexts [4,32].

The absence of an internal support network increases the risk of exhaustion and chronic stress. Socioeconomic barriers—limited finances, reduced work flexibility, and transportation difficulties—further complicate access to health resources.

Interventions must be adapted to the particularities of the single-parent context. Adapted family care plans can include flexible care pathways with evening or weekend sessions and referral to community services [3]. Psychosocial support and strengths-based interventions may foster resilience among sole caregivers [30]. Telehealth and hybrid models can support chronic disease management education, clarify doubts, and provide emotional support while reducing unnecessary travel and isolation [29,30].

The role of theoretical models in structuring interventions for single-parent families is also noteworthy. Models such as the Calgary Family Assessment Model and the Family Management Framework are valuable for mapping areas of strain and remaining resources in single-parent households [8,16]. The Family Resiliency Model guides interventions that strengthen problem-solving strategies and promote cohesion, which is essential when the caregiver lacks a partner for support [5]. Family Health Conversations provide a structured space for caregivers to express fears, strengthen ties with external support networks, and renegotiate care expectations [28].

### 4.2. Empowerment as an Outcome

The 29 included articles suggest that specialized Family Health Nursing interventions can enhance families’ capacity to manage their own health and family functioning. Studies using strengths-oriented interventions [31] or family pathways centered on members’ strengths [3] reported increased caregiver self-efficacy and greater confidence in handling crises and daily care routines. Structured family conversations and family-centered assessments may help single-parent families feel more actively involved in the therapeutic plan rather than positioned merely as recipients of instructions [16,28]. Telehealth interventions and hybrid programs were also associated with reduced caregiver burden and improved clarity about how and where to access community support, indicating empowerment through greater resource accessibility [29,30]. Beyond focusing on vulnerability, Family Health Nursing can highlight the resilience and potential for social transformation within single-parent families. These families often develop collective problem-solving strategies, such as informal support networks and intergenerational solidarity practices. By strengthening such networks, nurses can act as facilitators of more resilient communities. The Calgary Family Assessment Model proved particularly useful for mapping vulnerabilities and resources, while the Family Resiliency Model supported adaptive strategies consistent with Meleis’ Transitions Theory, which frames single parenthood as a life transition with the potential for both crisis and growth [5,16,17]. Capacity-building for Family Health Nurses in interventions specifically designed for single-parent families should therefore be a training priority.

Although the included studies were conducted in diverse sociocultural and health system contexts, the challenges identified—caregiver burden, social isolation, and barriers to accessing support—are consistently reported across primary health care settings. This suggests that the core principles underpinning Family Health Nursing interventions, particularly empowerment-oriented and family-centred approaches, may be transferable across contexts, while requiring contextual adaptation to local resources and cultural norms.

### 4.3. Single-Parent-Specific Findings

Although the integrative synthesis incorporated evidence derived from diverse family caregiving and health contexts, a focused analytical sub-synthesis was undertaken to identify findings specifically applicable to single-parent family experiences. Direct empirical evidence exclusively involving single-parent family samples was limited. However, through conceptual and contextual transferability analysis, several relational and empowerment-related processes emerged as particularly salient or intensified within single-parent family structures. Caregiving burden concentration was identified as a defining characteristic. In single-parent households, caregiving responsibilities are not distributed across partners, resulting in cumulative physical, emotional, and logistical demands. This concentration of care tasks amplifies stress exposure and reduces opportunities for respite and shared decision-making. Reduced relational support networks also emerged as a critical dimension. Single parents frequently rely on extended family members, community resources, or institutional support systems to compensate for the absence of a co-parental relational structure. The availability, accessibility, and quality of such support significantly influence coping capacity and family functioning. Heightened decision-making responsibility was another recurrent process. Single parents often assume sole responsibility for health-related decisions, care coordination, and system navigation. This intensified decisional burden may contribute to emotional strain but may also foster adaptive empowerment processes when adequate professional support is present. Across the reviewed evidence, relational empowerment processes were identified as both necessary and transformative. Therapeutic conversations, family-centred assessments, and strengths-based nursing interventions appear to facilitate resilience, resource mobilization, and self-efficacy among single-parent caregivers. Mapping these findings against the review research questions reinforces the need for Family Health Nursing approaches that are relationally attuned, structurally responsive, and empowerment-oriented when working with single-parent families in community and primary health care settings.

### 4.4. Evidence Gap in Family Health Nursing Support for Single-Parent Families

A central analytical finding emerging from this integrative review is the limited availability of empirical studies specifically addressing Family Health Nursing interventions targeted at single-parent families. Despite the increasing demographic prevalence of single-parent households and their well-documented social and health vulnerabilities, the scientific literature remains largely oriented toward broader family caregiving contexts, illness-related transitions, and generalized family support interventions. This absence of targeted intervention research necessitated the inclusion of conceptually transferable evidence derived from diverse family health and caregiving situations. While such evidence enabled the identification of relational, structural, and empowerment processes relevant to single-parent family experiences, it also revealed the insufficient development of tailored nursing frameworks addressing the unique realities of single-parent households. Single-parent families often face intensified caregiving demands, reduced relational support networks, economic vulnerability, and heightened decision-making burden, all of which require specific nursing assessment, intervention planning, and empowerment-oriented support strategies. However, these dimensions remain underexplored within intervention-based Family Health Nursing research. The identification of this evidence gap highlights an urgent need for future studies focused on the design, implementation, and evaluation of relational and community-based nursing interventions specifically tailored to single-parent family contexts. Strengthening this knowledge base is essential to advancing equitable, family-centred primary health care and supporting the development of specialized Family Health Nursing practice models.

### 4.5. The Relational Empowerment Model

The relational empowerment model was inductively derived from the thematic synthesis of the four principal domains identified in this integrative review: (1) caregiving burden and psychosocial vulnerability, (2) access to and coordination of community resources, (3) nurse–family relational processes, and (4) empowerment-oriented nursing interventions. The model conceptualises empowerment not as an isolated outcome, but as a dynamic and relational process emerging from the interaction between contextual vulnerability, concentration of caregiving responsibilities, therapeutic nurse–family engagement, and structured resource mobilisation strategies. These components do not operate linearly; rather, they interact in iterative and bidirectional ways across the structural, emotional, and relational dimensions of single-parent family life. Contextual vulnerability (e.g., socioeconomic constraints, social isolation, and reduced internal support networks) constitutes the structural backdrop within which single-parent families experience intensified caregiving demands. Caregiving concentration, characterised by the accumulation of parental, emotional, logistical, and decision-making responsibilities in one primary caregiver, functions as a central stress-amplifying mechanism. Nurse–family relational processes—such as family conversations, strengths-based dialogue, and culturally responsive engagement—serve as mediating mechanisms that transform vulnerability into adaptive capacity. Through these relational processes, families are supported in identifying internal strengths, accessing external resources, and strengthening decision-making competence. Resource mobilisation strategies, including coordinated referrals, telehealth integration, and community linkage, reinforce empowerment outcomes by enhancing accessibility, continuity of care, and self-efficacy.

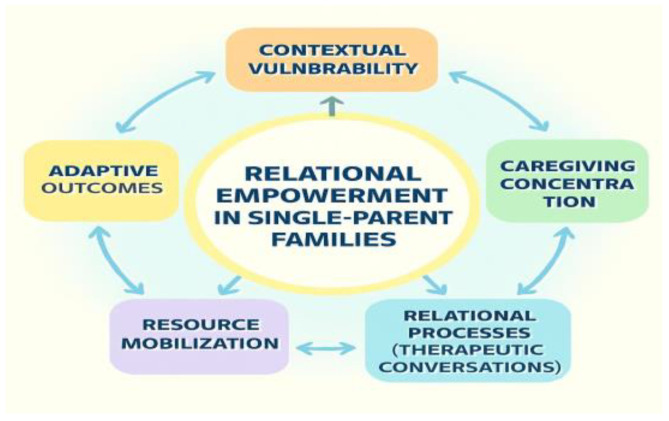



The analytical pathway linking synthesized evidence to model components is presented in Supplementary Table S1, which maps each component to its empirical support within the reviewed studies. This mapping clarifies the model’s grounding in the integrative synthesis and distinguishes empirically supported processes from theoretical extensions. Rather than proposing a prescriptive framework, the relational empowerment model offers a practice-informed, evidence-aligned structure to guide Family Health Nursing assessment, intervention planning, and professional education in community and primary health care contexts.

### 4.6. Suggestions for Practice and Research

Family Health Nurses should develop flexible, context-sensitive care pathways tailored to the structural and relational realities of single-parent families. This may include extended consultation hours, proactive linkage to financial and community support services, and structured family conversations focused on strengths and adaptive capacity. The integration of telehealth and hybrid care models may reduce social isolation, enhance accessibility, and improve continuity of care for single-parent caregivers facing logistical constraints. Future research should priorities evaluative, intervention-based, and cross-cultural studies specifically targeting single-parent family contexts in order to strengthen the empirical foundation of tailored Family Health Nursing practice. Nursing education programs should incorporate training in relational, empowerment-oriented, and culturally responsive family interventions.

## 5. Conclusions

This integrative review highlights the limited body of evidence specifically addressing Family Health Nursing interventions targeted at single-parent families in community and primary health care contexts. The synthesis therefore incorporated conceptually transferable evidence from broader family caregiving and vulnerability contexts to inform relational understanding. Findings underscore the complex relational, structural, and contextual challenges experienced by single-parent families, reinforcing the need for tailored nursing approaches grounded in partnership, empowerment, and resource mobilization. The proposed relational empowerment model emerges as a conceptual framework informed by the available evidence and designed to guide future research, intervention development, and clinical practice focused on strengthening Family Health Nursing support for single-parent families. Addressing the identified evidence gap represents a critical priority for advancing equitable and family-centered care.

## Figures and Tables

**Table 1:** Summary of Included Studies (n = 29).

No.	Transition Category (Meleis)	Family Life Cycle Stage (Calgary)	Challenges Identified	Reported Nursing Interventions	Theoretical Approach/Model Used	Single-Parent Specificity/Evidence Type
**1**	Health–Illness Transition	Family with an affected adult member	Adaptation to acquired brain injury; role reorganization	Family-centered care; emotional support	Family-Centered Care Pathway	transferable
**2**	Health–Illness Transition	Family with member in oncology treatment	High caregiver burden; caregiver anxiety and depression	Caregiver support interventions	Quantitative Longitudinal Study	transferable
**3**	Health–Illness Transition	Family with member with schizophrenia	Social stigma; weakened family resilience	Development of family resiliency model	Family Resiliency Model	transferable
**4**	Organizational Transition/New Family Role	Family managing a child’s chronic condition	Care coordination; lack of standardized measures	Use of Family Management Framework; review of family management measures	Family Management Framework	transferable
**5**	Theoretical Development Transition	Theoretical evaluation	–	Formulation of family health theory	Neuman’s Systems Model	transferable
**6**	Health–Illness Transition	Family with member in oncology treatment	Caregiver stress, anxiety, and depression	Strengths-oriented family intervention; reinforcement of internal resources	Strengths Oriented Intervention	transferable
**7**	Health–Illness Transition	Family with member with Alzheimer’s disease	Mental and physical overload; social isolation	Support strategies in family meetings; family education	Calgary Family Assessment Model	transferable
**8**	Role Transition/Parenting	Grandparents raising grandchildren	Task overload; lack of preparation for new roles	Emotional and practical support to grandparents; care education	Family Nursing Support	Transferable (non-parental caregiving)
**9**	Instrumental Assessment Transition	Family functioning evaluation	–	Cross-cultural validation of ICE EFFQ instrument	Psychometric Validation (ICE EFFQ)	Not transferable
**10**	Health–Illness/Adaptation Transition	Family with individual with Down syndrome	Adaptation to genetic condition; need for specialized support	Recommendations for family nursing practices	Scoping Review	transferable
**11**	Health–Illness Transition	Family with member with dementia	Communication conflicts; family anxiety	Structured family meetings; facilitated dialogue	Family Meetings Integrative Review	transferable
**12**	Methodological Transition	Evaluation of family typologies	Identification of family profiles for research	Latent Class Analysis to map typologies	Latent Class Analysis	transferable
**13**	Health–Illness Transition	Family of adults with cancer	Dysfunctional communication; overprotection	Communication mediation; training for open dialogue	Protective Buffering Integrative Review	transferable
**14**	Health–Illness Transition	Continued home care	Nurse insecurity in conducting family conversations	Training and supervision in family nursing conversations	Calgary Family Conversations	transferable
**15**	Family Formation Transition	LGBTQ adoptive families	Invisible losses; unacknowledged grief	Emotional support post-loss; grief therapy	Grief & Loss Framework	transferable
**16**	Health–Illness/End-of-Life Transition	Family in palliative care	Intense emotional distress; need for comprehensive care	Family-centered nursing actions at end-of-life	Family Focused Palliative Nursing	transferable
**17**	Sudden Loss Transition	Family after adolescent suicide	Trauma and family reorganization after sudden loss	Support groups and counseling; resilience strategies	Grounded Theory of Family Transformation	transferable
**18**	Health–Illness Transition	Family with member with neuromuscular disease	Daily management challenges; physical and emotional burden	Functional-focused interventions; practical counseling	Functional Assessment Tools	transferable
**19**	Structural Transition	Family with youth members	Barriers to access; institutional prejudice	Awareness programs; health professional training	Health Equity Framework	transferable
**20**	Health–Illness Transition	Family in home care for severe illness	Unmet emotional and practical needs	Review of home-visiting support practices	Scoping Review of Home-Based Support	transferable
**21**	Social Transition	Homeless families	Lack of resources; housing insecurity	Referral protocols; community support groups	Systematic Review of Homeless Families	transferable
**22**	Service Transition	Families in hybrid in-person/virtual program	Barriers to in-person access; social isolation	Hybrid care model; telehealth nursing	Hybrid Program Evaluation	transferable
**23**	Developmental Transition	Families with children in special needs schools	Need for specialized educational support; family stress	Emotional and physical support; educational guidance	Family Functioning Theory	transferable
**24**	Health–Illness Transition	Family caregivers of oncology patients	High caregiver burden; lack of social support	Stress management interventions; psychosocial support	Cross-Sectional Burden & Support Study	transferable
**25**	Social/Pandemic Transition	Families during COVID-19	Psychological impacts; mobility restrictions; isolation	Nurses’ perceptions and recommendations; adapted care guidelines	Descriptive Nursing Study	transferable
**26**	Service Transition	Culturally diverse families in home visiting	Language and cultural barriers; institutional mistrust	Sustained nurse home visiting; cultural competence training	Cultural Competence Framework	transferable
**27**	Health–Illness Transition	African American families caring for dementia	Specific emotional and economic challenges	Culturally sensitive support; community resources	Cultural Sensitivity in Family Care	transferable
**28**	Awareness Transition	General families	Need to resolve conflicts and improve cohesion	Regular Family Health Conversations	Family Health Conversations Model	transferable
**29**	Professional Role Transition	Families in all life cycle stages	Variable nurse attitudes; lack of preparation	Family attitude training programs; workshops	Survey of Nurse Attitudes	transferable

## Data Availability

No new data were created or analyzed in this study. Data sharing is not applicable to this article.

## Supplementary Materials

The following supporting information can be downloaded at: https://www.mdpi.com/article/10.3390/healthcare14081088/s1. Table S1. Methodological Quality Appraisal of Included Studies. Table S2. Methodological Characteristics of Included Studies.
