# Supplementary material for: Integrative Review of Family Health Nursing Support for Single-Parent Families: Evidence Gaps and Implications for a Relational Empowerment Model
Source: Healthcare (Basel). 2026 Apr 20;14(8):1088. doi: 10.3390/healthcare14081088 (PMC13116908; doi:10.3390/healthcare14081088)
Supplement: Supplementary file 1 [file healthcare-14-01088-s001.zip › suplementary file S1.pdf]

Supplementary File S1

Detailed Search Strategies

This supplementary file presents the complete database-specific search strategies used in this integrative review. The search strings include Boolean operators, truncations, phrase searching, and controlled vocabulary (MeSH and CINAHL Headings), reflecting the refined and expanded approach adopted following peer-review recommendations.

1. CINAHL (EBSCOhost)

Search Fields: Title / Abstract / Subject Headings

Search String:

("single parent*" OR "single mother*" OR "single father*" OR "lone parent*" OR "solo parent*" OR "one-parent famil*" OR "single-parent famil*" OR "single-parent household*")
AND
("family nursing" OR "family health nursing" OR "community nursing" OR "public health nursing" OR "primary health care nursing" OR "family-centered care" OR "nursing intervention*" OR "caregiver support")

Controlled Vocabulary:

- Single-Parent Family
- Family Nursing
- Primary Health Care
- Community Health Nursing

Limits Applied:

- Publication years: 2020–2025
- Languages: English, Portuguese, Spanish
- Peer-reviewed
- Full text available

2. PubMed / MEDLINE

Search Fields: Title / Abstract / MeSH Terms

Search String:

(("Single-Parent Family"[MeSH]) OR "single parent*" OR "single mother*" OR "single father*" OR "lone parent*" OR "solo parent*" OR "one-parent famil*")

AND
(("Family Nursing"[MeSH]) OR "family nursing" OR "family health nursing" OR "community nursing" OR "public health nursing" OR "primary health care" OR "family-centered care" OR "nursing intervention*" OR "caregiver support")

Filters Applied:

- Publication years: 2020–2025
- Languages: English, Portuguese, Spanish
- Humans
- Full text

3. Scopus

Search Fields: Title / Abstract / Keywords

Search String:

TITLE-ABS-KEY
("single parent*" OR "single mother*" OR "single father*" OR "lone parent*" OR "solo parent*" OR "one-parent famil*" OR "single-parent household*")
AND
TITLE-ABS-KEY
("family nursing" OR "family health nursing" OR "community nursing" OR "primary health care" OR "public health nursing" OR "family-centered care" OR "nursing intervention*" OR "caregiver support")

Limits Applied:

- Publication years: 2020–2025
- Document type: Articles / Reviews
- Languages: English, Portuguese, Spanish

4. LILACS

Search Fields: Title / Abstract / Descriptors (DeCS)

Search String:

("familia monoparental" OR "familia monoparental*" OR "madre soltera" OR "padre soltero")
AND
("enfermería familiar" OR "enfermería comunitaria" OR "atención primaria de salud" OR "cuidados centrados en la familia" OR "intervención de enfermería")

Descriptors (DeCS):

- Familia Monoparental
- Enfermería Familiar
- Atención Primaria de Salud

Limits Applied:

- Publication years: 2020–2025
- Languages: Portuguese, Spanish, English

Search Strategy Refinement Note

The search strategies presented above reflect the expanded and sensitivity-enhanced approach adopted following peer-review recommendations. Additional synonyms, truncations, and controlled vocabulary were incorporated to improve comprehensiveness and retrieval accuracy across databases.
