# Supplementary material for: Integrative Review of Family Health Nursing Support for Single-Parent Families: Evidence Gaps and Implications for a Relational Empowerment Model
Source: Healthcare (Basel). 2026 Apr 20;14(8):1088. doi: 10.3390/healthcare14081088 (PMC13116908; doi:10.3390/healthcare14081088)
Supplement: Supplementary file 1 [file healthcare-14-01088-s001.zip › suplementary file S2.pdf]

Table s1. Methodological Quality Appraisal of Included Studies

No.	Study Context	Study Design	Appraisal Tool Used	Overall Quality Level*
1	Acquired brain injury family care	Qualitative	JBI Qualitative Checklist	Moderate
2	Oncology caregiving burden	Quantitative longitudinal	CASP Cohort Checklist	High
3	Schizophrenia family resilience	Qualitative / Model development	JBI Qualitative Checklist	Moderate
4	Child chronic condition management	Integrative review	JBI Systematic Review Checklist	Moderate
5	Family health theory formulation	Theoretical study	Not applicable (conceptual)	—
6	Oncology strengths intervention	Quantitative intervention	CASP RCT / Intervention Checklist	High
7	Alzheimer's family support	Qualitative	JBI Qualitative Checklist	Moderate
8	Grandparents raising grandchildren	Qualitative	JBI Qualitative Checklist	Moderate
9	Family functioning instrument validation	Psychometric validation	JBI Analytical Cross-Sectional	High
10	Down syndrome family adaptation	Scoping review	JBI Scoping Review Checklist	Moderate
11	Dementia family meetings	Integrative review	JBI Systematic Review Checklist	Moderate
12	Family typologies mapping	Quantitative analytical	CASP Analytical Study Checklist	High
13	Cancer communication buffering	Integrative review	JBI Systematic Review Checklist	Moderate
14	Nurse family conversations training	Qualitative	JBI Qualitative Checklist	Moderate
15	LGBTQ adoptive families grief	Qualitative	JBI Qualitative Checklist	Moderate
16	Palliative family care	Qualitative	JBI Qualitative Checklist	Moderate
17	Adolescent suicide bereavement	Grounded theory	JBI Qualitative Checklist	High
18	Neuromuscular disease caregiving	Quantitative descriptive	CASP Cross-Sectional Checklist	Moderate
19	Youth structural inequities	Qualitative / Conceptual	JBI Qualitative Checklist	Moderate
20	Home-based severe illness care	Scoping review	JBI Scoping Review Checklist	Moderate
21	Homeless families support	Systematic review	JBI Systematic Review Checklist	High
22	Hybrid care program evaluation	Mixed methods	MMAT	High
23	Special needs school families	Qualitative	JBI Qualitative Checklist	Moderate
24	Oncology caregiver burden	Quantitative cross-sectional	CASP Cross-Sectional Checklist	High
25	COVID-19 family impacts	Descriptive quantitative	CASP Cross-Sectional Checklist	Moderate
26	Culturally diverse home visiting	Qualitative	JBI Qualitative Checklist	Moderate

27	African American dementia caregiving	Qualitative	JBI Qualitative Checklist	Moderate
28	Family health conversations	Qualitative intervention	JBI Qualitative Checklist	High
29	Nurse attitudes toward families	Quantitative survey	CASP Cross-Sectional Checklist	Moderate

*Quality levels were determined based on methodological clarity, sampling adequacy, data collection rigor, analytical transparency, and coherence between study aims and findings.

Table S2. Methodological Characteristics of Included Studies

No.	Study Design	Context	Population	Intervention / Focus
1	Qualitative	Community rehabilitation	Families of adults with acquired brain injury	Family-centered support; emotional coping
2	Quantitative longitudinal	Oncology care	Family caregivers of cancer patients	Caregiver burden and support interventions
3	Qualitative / Model development	Mental health	Families of individuals with schizophrenia	Family resilience processes
4	Integrative review	Pediatric chronic care	Families managing chronic childhood conditions	Family management frameworks
5	Theoretical study	Conceptual	Family systems	Development of family health theory
6	Quantitative intervention	Oncology care	Family caregivers	Strengths-based family intervention
7	Qualitative	Dementia care	Families of Alzheimer’s patients	Family meetings; education
8	Qualitative	Community caregiving	Grandparent caregivers	Parenting role adaptation support
9	Psychometric validation	Family assessment	General families	Instrument validation (family functioning)
10	Scoping review	Genetic conditions	Families of individuals with Down syndrome	Nursing practice recommendations
11	Integrative review	Dementia care	Family caregivers	Structured family meetings
12	Quantitative analytical	Family research	Diverse families	Family typology mapping
13	Integrative review	Oncology care	Family caregivers	Communication support interventions
14	Qualitative	Home care	Nurses working with families	Family conversation training
15	Qualitative	Adoption / LGBTQ families	Adoptive parents	Grief and loss support
16	Qualitative	Palliative care	Families at end of life	Family-centered palliative support
17	Grounded theory	Bereavement care	Families after adolescent suicide	Family transformation processes
18	Quantitative descriptive	Neuromuscular disease	Family caregivers	Functional caregiving support
19	Qualitative / Conceptual	Youth family health	Families facing inequities	Health equity awareness

20	Scoping review	Home care	Families managing severe illness	Home visiting support
21	Systematic review	Social vulnerability	Homeless families	Community referral systems
22	Mixed methods	Hybrid care programs	Families in telehealth programs	Hybrid nursing care models
23	Qualitative	Special education	Families of children with special needs	Family support and guidance
24	Quantitative cross-sectional	Oncology care	Caregivers	Psychosocial burden support
25	Quantitative descriptive	Pandemic context	Families during COVID-19	Adapted nursing care
26	Qualitative	Home visiting	Culturally diverse families	Cultural competence support
27	Qualitative	Dementia care	African American families	Culturally sensitive interventions
28	Qualitative intervention	Family nursing practice	General families	Family health conversations
29	Quantitative survey	Nursing workforce	Nurses working with families	Attitudes toward family care
